# A view on EGFR-targeted therapies from the oncogene-addiction perspective

**DOI:** 10.3389/fphar.2013.00053

**Published:** 2013-04-26

**Authors:** Rolando Perez, Tania Crombet, Joel de Leon, Ernesto Moreno

**Affiliations:** ^1^Center of Molecular ImmunologyHavana, Cuba; ^2^Biotech Pharmaceuticals Co. Ltd.Beijing, China

**Keywords:** EGFR, targeted therapy, oncogene addiction, combination therapy, cetuximab, erlotinib, nimotuzumab

## Abstract

Tumor cell growth and survival can often be impaired by inactivating a single oncogen– a phenomenon that has been called as “oncogene addiction.” It is in such scenarios that molecular targeted therapies may succeed. among known oncogenes, the epidermal growth factor receptor (EGFR) has become the target of different cancer therapies. So far, however, the clinical benefit from EGFR-targeted therapies has been rather limited. a critical review of the large amount of clinical data obtained with anti-EGFR agents, carried out from the perspective of the oncogene addiction concept, may help to understand the causes of the unsatisfactory results. In this article we intend to do such an exercise taking as basis for the analysis a few case studies of anti-EGFR agents that are currently in the clinic. There, the “EGFR addiction” phenomenon becomes apparent in high-responder patients. We further discuss how the concept of oncogene addiction needs to be interpreted on the light of emerging experimental evidences and ideas; in particular, that EGFR addiction may reflect the interconnection of several cellular pathways. In this regard we set forth several hypotheses; namely, that requirement of higher glucose uptake by hypoxic tumor cells may reinforce EGFR addiction; and that chronic use of EGFR-targeted antibodies in EGFR-addicted tumors would induce stable disease by reversing the malignant phenotype of cancer stem cells and also by sustaining an anti-tumor T cell response. Finally, we discuss possible reasons for the failure of certain combinatorial therapies involving anti-EGFR agents, arguing that some of these agents might produce either a negative or a positive trans-modulation effect on other oncogenes. It becomes evident that we need operational definitions of EGFR addiction in order to determine which patient populations may benefit from treatment with anti-EGFR drugs, and to improve the design of these therapies.

## INTRODUCTION

The concept of “oncogene addiction,” as enunciated by Weinstein (2002), arises from a series of experimental and clinical evidences showing that “cancer cells are often “addicted to” (that is, physiologically dependent on) the continued activity of specific activated or over-expressed oncogenes for maintenance of their malignant phenotype.” This concept provides a theoretical framework, whose bases stand both at the molecular and systems biology levels, that supports the targeted therapy approach in cancer treatment.

among known oncogenes, the epidermal growth factor receptor (EGFR) accounts for a significant number of the molecular targeting agents being used today in the clinic. These agents are either small-molecule tyrosine kinase inhibitors (STKIs), which block receptor signaling by interfering with aTP binding to the receptor (Quatrale et al., 2011), or monoclonal antibodies (mabs), which bind to the extracellular region of the receptor, inhibiting its dimerization and autophosphorylation (Schmitz and Ferguson, 2009).

Data supporting addiction in tumors have been gathered for a number of different oncogenes, as reviewed in Weinstein and Joe (2006). For the EGFR in particular, positive results in clinical trials with different antagonists have been considered as clinical evidences of oncogene addiction, even though the clinical benefits from the use of either mabs or STKIs have been rather limited. The question on which subset of cancer patients would be most benefited from these treatments is still under debate, and meanwhile the use of EGFR-targeted therapies in advanced cancer patients remains largely empirical.

a critical review of the large amount of clinical data obtained with different anti-EGFR agents, carried out from the perspective of the oncogene addiction concept, may help to better assess the phenomenon of “EGFR addiction” in human tumors, interpreting this addiction in a broad sense that comprises not only the receptor itself, but also its signaling pathway (Weinstein and Joe, 2008). a deeper understanding of this phenomenon would in turn contribute to a more rational, and therefore more effective clinical use of EGFR antagonists.

On the other hand, the concept of oncogene addiction itself needs to be interpreted on the light of the growing experimental evidences and newly emerging ideas. One of the hypotheses developed over the last years is based on the existence of a tumor cell hierarchy and suggests that tumorigenicity resides in only a small subpopulation of cancer stem cells (Driessens et al., 2012; Nguyen et al., 2012). Revising the concept of oncogene addiction under the premises of the cancer stem cell model becomes then a necessary theoretical exercise. In particular, we need to gain a deeper knowledge on the relevance of EGFR addiction for tumor initiating cells. another emerging body of evidences indicates that intracellular circuitries involving tumor metabolism and immunogenic cell death are connected to the EGFR signaling pathway (Weihua et al., 2008; Garrido et al., 2011a) and, therefore, they might be also involved in the phenomenon of EGFR addiction.

Results from the clinical use of different EGFR-targeted therapies lead to several relevant questions, namely: what are the molecular and cellular bases of intrinsic or acquired resistance? What would be the rationale for designing combinatorial therapies? What are the scenarios for chronic use of anti-EGFR agents? a better understanding of the complexity of the EGFR signaling network in human tumors would shed light on these questions and might contribute to establish operational definitions of “addiction-predictor” biomarkers. In this article we intend to do a hypothesis-generating exercise based on diverse pieces of clinical data, hoping to raise a debate that ultimately would promote both experimental and clinical research. a compendium of the main hypotheses discussed in the article is given in **Box 1**. The main goal at the end is to increase the effectiveness of available EGFR-targeted therapies in advanced cancer patients.

**Box 1. Summary of hypotheses.**

• Oncospecific treatment, radiotherapy and chemotherapy, reinforce EGFR addiction. Naïve addiction is reinforced by radiotherapy in primary tumors, while adaptive addiction arises as a resistance mechanism to radiotherapy and chemotherapy in recurrent disease.
• Only a few markers of EGFR addiction can be already defined, each one of them for a specific tumor type and a particular disease stage. No single universal predictor biomarker is likely to exist.
• The requirement of a higher glucose uptake by hypoxic tumor cells reinforces EGFR addiction.
• EGFR-targeted antibody therapy generates an anti-tumor T cell response. Chronic use of the EGFR antagonistic mab might be required to boost such response.
• The induction of stable disease with EGFR-targeted therapy is due, at least partially, to reversal of the malignant phenotype of cancer stem cells and inhibition of the epithelial-to-mesenchymal transition.
• Resistance mechanisms to EGFR-targeted therapies may provide clues for the design of combinatorial targeted therapies. Each tumor type, in a given disease stage, would have a predominant intrinsic resistance mechanism.

## CLINICAL IMPACT OF EGFR-TARGETED THERAPIES IN TWO CASE STUDIES: CETUXIMAB AND ERLOTINIB

Cetuximab is an EGFR-antagonistic antibody which is currently indicated for advanced head and neck and colorectal cancer (CRC). Cetuximab has been approved for the treatment of locally advanced squamous cell carcinoma of the head and neck (SCCHN) in combination with radiation therapy (RT; Bonner et al., 2006), for recurrent or metastatic carcinomas of the head and neck in combination with chemotherapy, and as monotherapy for recurrent or metastatic tumors of the head and neck progressing after platinum-based therapy (Vermorken et al., 2008). This antibody is also indicated for Kras mutation-negative (wild-type), EGFR-expressing, metastatic colorectal cancer (mCRC) in combination with FOLFIRI for first-line treatment (Van Cutsem et al., 2009). It is also indicated in combination with irinotecan for mCRC patients who are refractory to irinotecan-based chemotherapy (Cunningham et al., 2004), and as a single agent in CRC patients who have failed oxaliplatin and irinotecan-based chemotherapy (Jonker et al., 2007).

Erlotinib, on the other hand, is a STKI indicated for maintenance treatment in patients with advanced non-small cell lung cancer (NSCLC) whose disease has not progressed after four cycles of platinum-based first-line chemotherapy (Cappuzzo et al., 2010; Pérol et al., 2012); for treatment of advanced NSCLC after failure of at least one prior chemotherapy regimen (Shepherd et al., 2005); and as first-line treatment of patients with locally advanced pancreatic cancer, in combination with gemcitabine (Moore et al., 2007).

In spite of these marketing approvals, the impact of both cetuximab and erlotinib in terms of clinical benefit has been in general limited when evaluated for the overall populations included in the clinical trials.

Encouraging results were achieved with cetuximab in locoregionally advanced head and neck cancer. The median survival time (MST) was 49.0 months among patients treated with cetuximab and RT, versus 29.3 months among those treated with RT alone (Bonner et al., 2006). In a worse prognostic patient population, however, subjects with recurrent or metastatic carcinomas of the head and neck had a MST of 10.1 months if treated with cetuximab and platinum-based chemotherapy with fluorouracil (CTP), versus 7.4 months if treated with CTP alone (Vermorken et al., 2008). In the colorectal scenario, the addition of cetuximab to leucovorin, fluorouracil, and irinotecan (FOLFIRI) as first-line treatment for Kras wild-type mCRC resulted in a modest overall survival improvement (median, 23.5 versus 20.0 months; Van Cutsem et al., 2009). In another study with subjects whose disease had progressed within an irinotecan-based regimen, the combination of cetuximab and irinotecan yielded a MST of 8.6 months, versus 6.9 months for the cetuximab monotherapy group (Cunningham et al., 2004). Finally, patients with EGFR-expressing CRC, who had been previously treated with fluoropyrimidine, irinotecan and oxaliplatin, had a median overall survival of 6.1 months if treated with cetuximab, and 4.6 months if receiving supportive care alone (Jonker et al., 2007).

Cetuximab has been evaluated also in other scenarios without ending up in marketing approvals. This was the case for the FLEX (First-Line ErbituX in lung cancer) study, which compared cisplatin and vinorelbine plus cetuximab with cisplatin and vinorelbine alone in the first-line treatment of 1125 patients with EGFR-expressing, advanced NSCLC. The study showed that the addition of cetuximab to chemotherapy improved overall survival only from 10.1 to 11.3 months (Pirker et al., 2009).

Clinical results with erlotinib have evidenced a similar limited impact. In a study carried out with patients with stage IIIB or IV NSCLC who previously went through one or two chemotherapy regimens, the overall survival was 6.7 months for the group treated with erlotinib, versus 4.7 months for the placebo group (Shepherd et al., 2005). In the SaTURN trial for NSCLC patients with non-progressive disease following first-line platinum-doublet chemotherapy, the median progression-free survival (PFS) was only slightly longer with erlotinib than with placebo: 12.3 versus 11.1 weeks (Cappuzzo et al., 2010).

In a more recent clinical trial, patients with IIIB/IV NSCLC without tumor progression after four cycles of cisplatin–gemcitabine were randomly assigned to observation or to gemcitabine or erlotinib. as compared to the observation group, PFS was prolonged by gemcitabine from 1.9 to 3.8 months, and to 2.9 months by erlotinib (Pérol et al., 2012). Thus, both maintenance strategies resulted in poor improvements in overall survival. Lastly, in the pancreatic setting, patients with advanced tumors received standard gemcitabine plus erlotinib or gemcitabine plus placebo. The overall survival was prolonged in the erlotinib/gemcitabine arm from 5.91 to 6.24 months, which represents only a 13-day advantage in overall survival (Moore et al., 2007). It should be noted that in all the mentioned studies with erlotinib no biomarker was used for patient selection.

How should we interpret the above described results? Shall we conclude that EGFR-targeted therapies have a minor impact on survival in advanced cancer patients? a more detailed analysis of the clinical data, however, leads to an alternative interpretation, namely, that EGFR-targeted therapies may benefit only a subpopulation of patients – those whose tumors show EGFR oncogene addiction.

## HIGH-RESPONDER PATIENTS PROVIDE CLINICAL EVIDENCES OF EGFR ONCOGENE ADDICTION

The 1.2 month increase in MST observed for the combination of cetuximab with chemotherapy in the FLEX trial, conducted in EGFR-expressing NSCLC patients, was obtained from the analysis of the intent-to-treat population. With this type of data analysis, the Kaplan–Meier survival curves for the treatment and control arms start to separate after 7 months, a time point at which both arms had about a 60% survival rate (hazard ratio (HR) = 0.871, *p* = 0.044). However, when the analysis was carried out separately for the low and high EGFR-expression tumors, the curves showed quite different outcomes. For low EGFR-expression tumors no difference was found between the treatment and control arms (HR = 0.99, *p* = 0.88), whereas for high EGFR-expression tumors there is an evident early separation of the survival curves (approximately after 4 months) and a significant survival advantage for the group receiving cetuximab plus chemotherapy (HR = 0.73, *p* = 0.011; Pirker et al., 2009).

a similar phenomenon of time-delayed separation of the PFS Kaplan–Meier curves was observed with erlotinib used as maintenance therapy after first-line chemotherapy in NSCLC patients. In this case, stratification according to EGFR-mutation status gives rise to two subpopulations with quite different clinical responses to erlotinib (Pérol et al., 2012). Likewise, in the SaTURN trial, a profound predictive effect on PFS of erlotinib relative to placebo was observed in the EGFR mutation-positive subgroup (HR = 0.1, *p* = 0.001), whereas a lower clinical benefit was observed for the wild-type EGFR subgroup (HR = 0.78, *p* = 0.0185; Cappuzzo et al., 2010). In the study conducted by Shepherd et al. (2005), the likelihood of a response to erlotinib among patients with NSCLC was higher among patients with adenocarcinoma [objective response rate (ORR) = 13.9% for erlotinib, versus 4.1% for placebo], and therefore adenocarcinoma was associated with survival benefit. Interestingly, in NSCLC patients, EGFR-activating mutations are found mostly in those with adenocarcinomas (Rosell et al., 2009). Overall, activating mutations in the tyrosine kinase domain of EGFR seem to increase sensitivity to erlotinib in advanced NSCLC patients in terms of response rate and PFS.

In patients with locoregionally advanced head and neck cancer, the combination of cetuximab with radiotherapy conferred roughly a 20-month increase in MST, as quoted above. It should be noted, however, that this advantage was limited to patients with oropharynx tumors, which were irradiated with a regimen including concomitant boost (Bonner et al., 2006). It has been reported that high EGFR expression correlates with resistance to radiotherapy (Jedlinski et al., 2013), therefore blocking the EGFR signaling would induce radio-sensitivity. We would speculate that the opposite effect also takes place, i.e., under RT tumors with high EGFR expression, such as oropharynx tumors (Luedke et al., 2012), may become even more EGFR-addicted.

In mCRC cetuximab in combination with FOLFIRI for first-line treatment provides a therapeutic benefit in a patient subpopulation having EGFR-positive tumors (as defined based on immunohistochemical evidence of EGFR expression) and wild-type Kras gene expression, for whom the Kaplan–Meier progression-free and overall survival curves show an early separation (Van Cutsem et al., 2009). Thus, EGFR expression, although a necessary condition, is not sufficient to ensure therapeutic benefit. This is explained by the fact that Kras mutations that turn downstream signaling independent of EGFR activation provide an alternative, escape route to satisfy the addiction to the EGFR signaling pathway. It is tempting to speculate that the relative abundance of tumor cells with activating mutations in the EGFR or in Kras that is found in some tumors, e.g., mCRC, may result from a Darwinian process under the selective pressure exerted by first-line chemotherapy, with higher probabilities of occurrence in adenocarcinomas. another interesting phenomenon observed in the clinic in mCRC is that 20% of the patients that are refractory to irinotecan respond to the combinatorial therapy of cetuximab plus irinotecan (Cunningham et al., 2004). a plausible interpretation is that in these patients, resistance to irinotecan is associated to an increased addiction to the EGFR, which becomes impaired upon cetuximab treatment.

## EVIDENCES OF ONCOGENE ADDICTION IN EGFR-OVEREXPRESSING TUMORS FROM OUR CLINICAL EXPERIENCE WITH NIMOTUZUMAB

Nimotuzumab (also known as h-R3) is a humanized anti-EGFR mab (Mateo et al., 1997) developed at the Center of Molecular Immunology in Havana, Cuba. Since 1998, nimotuzumab has been extensively tested in 28 completed clinical trials in Cuba (10), Canada (4), US (1), China (2), Germany (4), India (4), Japan (2), and South Korea (1). During 2012, 29 clinical trials were ongoing in Cuba (8) and other 10 countries: Brazil (4), China (8), Germany (1), India (2), Indonesia (1), Japan (2), Mexico (1), and Singapore (2). Nine of them correspond to phase III or phase IV trials. It is estimated that roughly 30,000 patients have been treated with the antibody worldwide. Currently, nimotuzumab is indicated for the treatment of patients bearing advanced head and neck, nasopharyngeal tumors, adult high grade glioma, children glioma, and advanced esophageal cancer; and has been registered in more than 30 developing countries, including Brazil, China, and India.

Several pieces of information have been published so far indicating that nimotuzumab has a better clinical effect in tumors that over-express the EGFR. Rodríguez et al. (2010) conducted a phase II clinical trial in 106 advanced SCCHN patients, mostly unfit for chemo-radiotherapy, to assess the efficacy of nimotuzumab in combination with radiotherapy. In the intent-to-treat analysis, the median survival for patients in the nimotuzumab and control (receiving RT and placebo) groups were 12.5 and 9.5 months, respectively. EGFR expression was evaluated in tumor biopsies from 55 patients before enrollment in the trial and separate survival analyses were done for patients showing at least a weak EGFR expression and for EGFR-negative subjects. For EGFR-positive patients (as determined by immunohistochemical staining using a qualitative scale), a significant improvement in MST was observed within the group treated with nimotuzumab as compared to those from the control group (16.5 versus 7.2 months, *p* = 0.0038), whereas no significant advantage was seen for EGFR-negative patients. a similar behavior was observed in a phase IIB clinical trial where 92 treatment-naïve patients with advanced head and neck squamous cell carcinoma received standard therapy either with or without nimotuzumab (Basavaraj et al., 2010). Here also, EGFR expression showed a significant correlation with patient survival in patients treated with nimotuzumab and chemoradiation (*p* = 0.02).

In another study, 63 patients with non-resectable, esophageal cancer of epithelial origin received nimotuzumab in combination with radiation and chemotherapy, or radiation and chemotherapy alone (Ramos-Suzarte et al., 2012). The objective response rates per protocol were 47.8 versus 15.4% (*p* = 0.014) for the nimotuzumab and control groups, respectively, while the disease control rates (DCRs) were 60.9 and 26.9% (*p* = 0.017). Tumor EGFR expression at baseline was evaluated for 18 patients (13 from the nimotuzumab arm and 5 from the control arm). The EGFR expression was classified as high in 10 out of the 13 patients treated with nimotuzumab (77%) and in 4 out of the 5 control subjects (80%). For patients that over-express the EGFR, the objective response rate was 60% and DCR was 80%, which improves on the response and DCR seen in the per-protocol population.

Finally, in a phase II clinical trial conducted by Kim et al. (2011) in gastric cancer patients refractory to 5Fu-based therapy, no significant PFS and overall survival benefit was found in the intent-to-treat population. as in the previous studies, the baseline EGFR expression was evaluated in a group of patients and, again, PFS and overall survival showed a large trend toward survival benefit for those subjects with medium and high EGFR expression.

In the particular case of nimotuzumab, the relationship between the levels of EGFR expression and clinical benefit may have an explanation at the molecular level, based on the “intermediate” affinity of this antibody (Crombet et al., 2004). SPR/biacore experiments showed that the nimotuzumab Fab fragment has a KD of the order of 10^-^^8^ M; that is, an order of magnitude weaker than cetuximab Fab’s KD (Talavera et al., 2009). In *in vitro* experiments, binding of nimotuzumab and subsequent inhibition of the EGFR phosphorylation was detected only for tumor cell lines with medium or high levels of EGFR expression (10^4^ receptors per cell or higher). Furthermore, the Fab fragments bound only to a431 cells – those with the highest EGFR expression level. In contrast, cetuximab Fab fragments were able to bind to tumor cells with lower EGFR expression levels (Garrido et al., 2011b). These results sustain the idea that nimotuzumab requires bivalent binding for stable attachment and therefore would bind preferentially to tumor cells having a medium or high surface density of EGFR molecules. They also explain the low toxicity profile showed by this antibody in the clinical practice. Several other properties of nimotuzumab that may contribute to its clinical effects have been discussed in a recent review (Perez et al., 2011).

Nimotuzumab’s low toxicity profile has made possible the intent of using an anti-EGFR agent in continuous, long-term treatment (lasting several months, and a few years in several cases), which has so far been administered to a few hundred advanced cancer patients (Perez et al., 2011). For example, in the prospective clinical study by Saurez et al. (2009) that included 22 pediatric patients with brain tumors, 10 of these patients received around 30 or more nimotuzumab doses, which in terms of treatment time corresponds to 1 year or even longer. The median overall survival was increased from barely a few weeks to 19 months.

The frequently observed disease stabilization and increase of overall survival resulting from such chronic treatment suggest that certain level of oncogene addiction is maintained in those tumors during long periods. Disease stabilization might result from controlling effects on cancer stem cells and modulation of the malignant phenotype, and/or enhancement of the natural anti-tumor immune response, as will be discussed further below.

## HOW TO PREDICT EGFR-ONCOGENE ADDICTION IN THE CLINICAL SETTING?

### “NAÏVE” VERSUS “ADAPTIVE” ONCOGENE ADDICTION

The phenomenon of EGFR oncogene addiction seems to have several ways of manifesting in the clinic. a first difference can be observed between patients that are being subjected to first-line therapy and patients that have become refractory to previous therapies. In the first case we would say that we are in the presence of a “naïve” oncogene addiction, i.e., an addiction that arises during tumor progression, whereas the second case would correspond to an “adaptive” addiction, which develops as a resistant mechanism driven by chemo- or radiotherapy.

Naïve EGFR addiction reveals in locally advanced SCC (for example, SCCHN, and the SCC histological subtype of NSCLC), mostly in tumors having EGFR over-expression, as evidenced by the clinical responses observed in patients upon treatment with anti-EGFR agents (Bonner et al., 2006; Pirker et al., 2012). Emergence of adaptive EGFR addiction is observed for tumors that are refractory to chemotherapeutic agents, which then respond to the combination of chemotherapy with an anti-EGFR agent. This adaptive addiction is found even in tumors with low to medium EGFR expression (for example, CRC, and gastric and pancreas tumors). In this regard, it is worth noting that EGFR expression is usually assessed by immunohistochemistry in samples coming from primary tumors, and less often from metastases.

### GENETIC MODIFICATIONS LEADING TO EGFR ADDICTION

The investigations on the EGFR and its ligands have been closely related to oncology since their very first steps in the 1980s. among the early discoveries disclosing this relationship are the finding of transforming growth factor alpha (TGFα) as part of an autocrine loop leading to cell malignant transformation (Sporn and Todaro, 1980); the high sequence homology found between the EGFR and the retroviral oncogenic protein called v-ERBB (Downward et al., 1984); the increased EGFR expression observed in human squamous cell lung cancers (Hendler and Ozanne, 1984), and the subsequent finding of EGFR aberrant expression and gene amplification in a human tumor cell line (Ullrich et al., 1984). Today, EGFR over-expression is a hallmark in molecular oncology. It is found in many different types of epithelial derived tumors, often owing to gene amplification (Yarden and Pines, 2012). Gene transcription regulation by microRNas, such as miR-128b, is another cause of EGFR over-expression (Weiss et al., 2008). miR-128b loss-of-heterozygosity has been found in NSCLC patients and has been shown to be positively correlated with clinical response and survival following gefitinib treatment (Weiss et al., 2008). Several other EGFR genetic alterations with oncogenic potential have been reported; for example, deletion mutants in glioblastoma multiforme (GBM; deletion of exons 2–7, denoted EGFRvIII) and in NSCLC (exon 19), and activating kinase domain mutations in NSCLC, for example, the leucine-to-argine substitution at position 858 (L858R; Rosell et al., 2007).

### RESISTANCE MECHANISMS TO EGFR-TARGETED THERAPIES ARE A MANIFESTATION OF ONCOGENE ADDICTION

Treating an EGFR-addicted tumor with an anti-EGFR agent creates a selection pressure favoring the survival of those cells that are able to avoid the effect of the drug; that is, those cells that find an escape mechanism to satisfy their addiction. In one type of mechanism, the EGFR evades the drug via mutations that impair drug binding or enhance receptor functioning. The former is the case of the S492R mutant, which confers resistance to cetuximab since the mutation is located in its binding epitope on the EGFR external domain (Montagut et al., 2012); whereas the later is the case of the T790M mutant (mutation in the kinase domain), which confers resistance to gefitinib or erlotinib in lung adenocarcinomas (Pao et al., 2005) by stabilizing the active tyrosine kinase conformation and enhancing aTP binding (Yoshikawa et al., 2013).

In a second type of resistance mechanism, the effect of the drug is evaded by making irrelevant the function of the EGFR itself, while ensuring downstream signaling via the PI3K/aKT or RaS/RaF/MEK/ERK pathways. In CRC patients, KRaS mutations that constitutively activate this enzyme produce a primary resistance to EGFR-targeted mabs (Lièvre et al., 2006). activation of compensatory signaling pathways, like the PI3K/akt pathway, is also a way to bypass a blocked EGFR. Inactivating mutations in PTEN (phosphatase and tensin homolog), which has a tumor suppressor function, produce such activation of the PI3K/akt survival pathway, causing resistance to TKIs and anti-human epidermal growth factor receptor-2 (anti-HER2) antibodies (Garrett and arteaga, 2011). Other members of the ERBB family may play important roles in activating compensatory signals; for example, acquired resistance to cetuximab in CRC has been linked to activation of ERBB2 signaling (Yonesaka et al., 2011), while acquired resistance of lung tumors to gefitinib involves activation of the PI3K pathway through ERBB3 (Engelman et al., 2007).

### EVALUATION OF RESPONSE PREDICTOR BIOMARKERS

Different biological substrata might support EGFR addiction in different stages of the disease and in different tumor localizations, and in consequence, different response predictor and treatment selection biomarkers would be needed. In 2009 the FDa updated the registration labels for panitumumab and cetuximab, specifying that they are approved for the treatment of CRC tumors expressing wild-type KRaS. This was indeed a milestone in the way toward the development of personalized EGFR-targeted therapies. In addition to the KRaS mutation status, it has been reported that expression of epiregulin and amphiregulin is a response predictor in metastatic CRC patients treated with cetuximab (Khambata-Ford et al., 2007). another case of successful use of a biomarker has been the assessment of activating EGFR mutations in NSCLC (for example, L858R), which predicts response to STKIs. These mutations are more frequent in the adenocarcinoma subtypes of NSCLC (Rosell et al., 2009).

In spite of the variety of scenarios, EGFR over-expression seems to be a common hallmark of EGFR oncogene addiction, although the existent disagreements concerning the appropriate method for measuring and classifying the level of EGFR expression have yielded contradictory results and discrepancies in regard to its relevance. Other practical difficulties may hinder the use of this biomarker, for example, determining the presence of adaptive EGFR addiction would demand the evaluation of biomarkers in refractory metastatic lesions, which is not always feasible. In general, we need a more systematic and comprehensive analysis of both EGFR signaling-activating and resistance-inducing mutations, as well as an analysis of the activation of compensatory signaling pathways that may result upon treatment with EGFR-targeted agents, in order to validate in the clinical setting the already known or suspected predictor biomarkers, and to define new ones.

## EGFR ONCOGENE ADDICTION MAY REFLECT THE INTERCONNECTION OF SEVERAL CELLULAR PATHWAYS

### THE NEED FOR AN INCREASED GLUCOSE METABOLISM MAY REINFORCE EGFR ONCOGENE ADDICTION

It is well-known that EGFR activation upon ligand binding induces survival signaling through the PI3K/akt pathway, which upregulates anti-apoptotic factors (Engelman, 2009). Therefore abrogation of EGFR activation by targeting either the ectodomain or the kinase intracellular domain favors the induction of apoptotic cell death. In a recent report, a different mechanism to induce cell death was uncovered. The EGFR was shown to prevent autophagic cell death by maintaining the intracellular glucose level, most likely through stabilizing interactions with the sodium/glucose co-transporter 1 (SGLT1; Weihua et al., 2008). abrogating the EGFR gene expression by small interfering RNa (siRNa) resulted in loss of SGLT1, leading to a decrease in the intracellular glucose level. Since tumor micro-environment is characterized by hypoxia and nutrient starvation, pathways that guarantee an active glucose transport become critical for tumor cell survival. In this context, the stabilizing effect exerted by the EGFR on the sodium/glucose co-transporters might contribute to reinforce the addiction of tumor cells to this oncogene. Recent clinical studies have demonstrated that co-expression of EGFR and SGLT1 is associated with differentiation and prognosis of human tumors (Guo et al., 2011; Hanabata et al., 2012). Moreover, ionizing irradiation of human lung adenocarcinoma cells increased SGLT1 expression as a survival mechanism that depends on EGFR signaling (Huber et al., 2012).

The molecular details of the interaction between the EGFR and SGLT1 remain unknown. It is known, however, that the stabilizing effect on SGLT1 depends on the extracellular region of the EGFR, while being independent of the activity of the tyrosine kinase domain. In a plausible model, these molecules would interact, either directly or indirectly, in the context of a macromolecular assembly at the cell membrane. Specific anti-EGFR antibodies might then affect such interactions by increasing receptor internalization, thus destabilizing the macromolecular assembly and leading to a down-regulation of the SGLT1 expression. Following the above reasoning, tumors with high EGFR expression and showing positive positron emission tomography (PET) images would be highly sensitive to EGFR antagonistic antibodies. Here, nevertheless, we have to take into account that 2-(18F)-2-deoxy-D-glucose (the glucose analog used in PET imaging) is a poor substrate for SGLT1 (Wright et al., 2011) and, therefore, PET studies with this tracer would not be measuring the glucose uptake via the SGLT1 co-transporter.

### FUNCTIONAL INHIBITION OF AN “ADDICTIVE” ONCOGENE MAY INDUCE IMMUNOGENIC CELL DEATH

Experiments using a murine EGFR (mEGFR)-antagonistic antibody in a syngeneic preclinical model demonstrated that the anti-metastatic effect produced by treatment with this mab is mediated by T cells, since depletion of CD4^+^ and CD8^+^ T cells abrogated the anti-tumor effect (Garrido et al., 2007). In a different experimental setting, mice immunized with the extracellular domain of the mEGFR developed a strong antibody response with high EGFR-antagonistic activity, which resulted in reduction of lung metastases (Ramírez et al., 2006, 2008). This anti-metastatic effect of the mEGFR vaccine also was abolished by *in vivo* depletion of the CD8+ T lymphocyte subpopulation (aguiar alpizar et al., 2012). Furthermore, it was demonstrated that an anti-EGFR antibody, but not a STKI, promotes an immunogenic cell death in a Lewis lung carcinoma model, involving induction of a CTL response *in vivo* (Garrido et al., 2011a). Remarkably, the immunogenic effect found in these experiments was independent of the effector functions of the antibody, since the Fab’(2) fragments were shown to induce immunogenic apoptosis. T cell responses have been measured and have been found to be relevant also in other models of oncogene addiction, as in a recent study demonstrating that CD4+ T cells are required for tumor regression upon inactivation of the MYC or BCR–aBL oncogenes in mouse models of T cell acute lymphoblastic lymphoma and pro-B cell leukemia, respectively (Rakhra et al., 2010).

all together, these experimental results suggest that functional inhibition of an “addictive” oncogene may trigger an immunogenic cell death which activates a T cell-mediated anti-tumor response, although as evidenced in the above described experiment with a STKI, not every inhibition mechanism would yield this effect. Enhancement of the natural anti-tumor immunity, if sustained, might contribute to a long-lasting control of the disease by counteracting the tumor resistance to the targeted therapy.

### EGFR ONCOGENE ADDICTION MAY BE RELEVANT FOR TUMOR INITIATING CELLS

Different lines of experimental evidences point to the existence of EGFR addiction in tumor initiating cells derived from neural tissue. GBM derived tumor initiating cells that express EGFR display the most malignant functional and molecular phenotype. Modulation of EGFR expression in these cells by gain- and loss-of-function strategies enhances or reduces their tumorigenic ability, suggesting that EGFR plays a fundamental role in gliomagenesis (ayuso-Sacido et al., 2010; Mazzoleni et al., 2010). Noteworthy, PI3K and the mammalian target of rapamycin (mTOR) are involved in the epidermal growth factor (EGF)-mediated maintenance of neural progenitor cells, supporting their self-renewal capacity and non-differentiated state (Sato et al., 2010). Indeed, brain cancer stem cells are preferentially sensitive to akt signaling inhibition, which reduces the number of viable cells relative to matched non-stem cancer cells. The described mechanism suggests a preferential induction of apoptosis and a suppression of neurosphere formation, driving an increase in survival of immune-compromised mice bearing human glioma xenografts (Eyler et al., 2008). GBM CD133-positive tumor initiating cells have proven to be radio-resistant and most likely are the source of tumor recurrence after radiation (Bao et al., 2006). It has been shown, however, that combination of anti-EGFR antibodies, namely cetuximab and nimotuzumab with radiotherapy reduces the number of CD133-positive tumor initiating cells (Diaz Miqueli et al., 2009). This suggests that radiation-based therapy reinforces the EGFR oncogene addiction of neural cancer stem cells, adding a rationale for combining anti-EGFR antibodies and radiotherapy to treat brain tumors.

The relevance of the EGFR signaling pathway in the survival, maintenance, and function of cancer stem cells have been demonstrated also for head and neck and breast tumors (Hardy et al., 2010; abhold et al., 2012; Yan et al., 2012). activation of EGFR in head and neck SCC *in vitro* resulted in increased tumor sphere formation, while treatment with gefitinib, also *in vitro*, decreased the capacity of putative cancer stem cells to invade and made them more sensitive to cisplatin-induced death (abhold et al., 2012). In breast cancer cells, EGFR signaling activation can induce epithelial to mesenchymal transition (EMT), favoring invasion and metastasis along with increased expression of genes associated with self-renewal, increased percentage of stem-like cells, *in vitro* sphere formation and *in vivo* tumor growth (Del Vecchio et al., 2012; El-Haibi et al., 2012). In aggressive inflammatory breast cancer, inhibition of the EGFR reversed the mesenchymal phenotype of cancer cells to a less aggressive and potentially more chemotherapy-sensitive epithelial phenotype (Zhang et al., 2009).

Modulation of the malignant phenotype resulting from EGFR inhibition can be seen as a manifestation of the phenomenon of EGFR oncogene addiction, which in this case has a particular translational relevance because we may infer from it that chronic use of EGFR-targeted therapy would have a controlling effect on EGFR-addicted metastases. Our clinical experiences using nimotuzumab for long-term treatment of advanced cancer patients, as discussed above, give certain support to this hypothesis.

## COMBINATORIAL TARGETED THERAPY CAN MODULATE ONCOGENE ADDICTION: FRIEND OR FOE?

In combining targeting agents different strategies can be followed. One approach is to combine agents acting either on the same or on different targets, but in the same signaling pathway. another approach is to combine agents acting on targets in different pathways or cellular mechanisms. The rationale behind the first approach would be that cancer cells are addicted to specific signaling pathways rather than to a single oncogene, therefore the combination of agents acting on the same pathway may have a stronger inhibitory effect (Weinstein and Joe, 2006). On the other hand, signaling pathways have evolved to adapt to rare mutations, therefore they would be more sensitive to multiple hits (Yarden and Pines, 2012). The second approach may, theoretically, involve cellular pathways related to the complexity of the tumor biology; for example, targeting molecules involved in cancer-related inflammation (Mantovani et al., 2008) and tumor metabolic re-programming (Kroemer and Pouyssegur, 2008). But as we discussed above, we still lack knowledge for the rational design of such combinatorial targeted therapies. To date, the combination studies performed with anti-EGFR drugs, even though guided by these strategies, have at the same time been biased by the available therapeutic agents.

### DOES MULTIPLE-TARGETING OF EGFR IMPAIR RESISTANCE INDUCTION?

The combination of an anti-EGFR antibody with a STKI, namely cetuximab and erlotinib, has been attempted only in a couple of early phase trials designed to optimize the dose and treatment schedule. In one of these studies, 19 patients with lung adenocarcinoma and clinically defined acquired resistance to erlotinib were treated with 100 mg erlotinib daily, along with cetuximab every 2 weeks in three escalating dose cohorts (250–500 mg/m^2^). at these doses and treatment schedule no radiographic responses were seen, so the authors concluded that the combination had no significant activity in patients with acquired resistance to erlotinib (Janjigian et al., 2011). In the second study, 22 patients with advanced solid malignancies (including 14 patients with NSCLC) who had failed standard chemotherapies received escalating doses of cetuximab (100–250 mg/m^2^ i.v. weekly) in combination with a fixed dose of erlotinib (150 mg daily, orally) until disease progression or unacceptable toxicity (Guarino et al., 2009). The authors concluded that dual EGFR inhibition with cetuximab and erlotinib was feasible, but no conclusions were obtained on response rates and other clinical endpoints. The question on whether combining different EGFR antagonists may impair resistance induction, remains to be answered.

### SIMULTANEOUS TARGETING OF EGFR AND VEGF IN THE CLINIC HAS BEEN DISAPPOINTING

Targeting agents directed at the vascular endothelial growth factor (VEGF), such as bevacizumab, and to the EGFR, such as cetuximab and panitumumab, have become part of the standard treatment of mCRC. Earlier experimental work demonstrated that acquired resistance to anti-EGFR antibodies can be mediated by constitutive up-regulation of VEGF gene expression (Viloria-Petit et al., 2001; Crombet-Ramos et al., 2002), suggesting that simultaneous targeting of EGFR and VEGF may impair resistance induction. However, several recent phase III trials have shown a detrimental effect from adding an anti-EGFR antibody to standard chemotherapy plus bevacizumab. In the CaIRO trial (Tol et al., 2009), patients with previously untreated mCRC were randomized to capecitabine, oxaliplatin, and bevacizumab (CB regimen) or to the same regimen plus weekly cetuximab (CBC regimen). The results of this trial were disappointing – the median PFS was 10.7 months in the CB group versus 9.4 months in the CBC group (*p* = 0.01), and the quality-of-life scores were lower in the CBC group. The overall survival and response rates did not differ significantly in the two groups. In another trial, advanced CRC patients were randomized to either the combination of bevacizumab, leucovorin, and 5-fluorouracil (5-FU), or the same combination plus cetuximab. The 12-month PFS for the two groups were 45 versus 32%, ORR – 52 versus 41%, DCRs – 87 versus 83%, and the median overall survival times were 21 versus 19.5 months. In summary, the combination including cetuximab was not superior. The conclusion after these trials is that cetuximab and bevacizumab should not be used concurrently in metastatic CRC (Saltz et al., 2012).

Similar results were obtained in the phase III Panitumumab advanced Colorectal Cancer Evaluation (PaCCE) trial, that investigated panitumumab added to a regimen combining bevacizumab with chemotherapy. This trial resulted in unacceptable toxicities in the investigation arm and no differences in efficacy, leading to discontinuation of the study. Overall, the addition of panitumumab reduced both the median PFS and the median overall survival.

Combining bevacizumab with a STKI has been also assayed. In a phase III trial, patients with metastatic pancreatic adenocarcinoma were randomly assigned to gemcitabine, erlotinib, and bevacizumab, or gemcitabine and erlotinib. adding bevacizumab to gemcitabine–erlotinib significantly improved PFS (HR = 0.73; 95% CI, 0.61 to 0.86; *p* = 0.0002). However, the differences in MST (7.1 versus 6.0 months for the bevacizumab and placebo arms, respectively; HR = 0.89, *p* = 0.2087), were not statistically significant (Van Cutsem et al., 2009).

Not all the combination experiences have been negative, though. In particular, some encouraging results have been seen in the NSCLC setting when combining bevacizumab and erlotinib. In the BeTa phase III trial (Herbst et al., 2011), patients with recurrent or refractory NSCLC that had failed a first-line treatment were allocated to receive erlotinib plus bevacizumab or erlotinib plus placebo. The median overall survival did not differ between the two groups (9.3 versus 9.2 months), but PFS seemed to be longer in the bevacizumab group (3.4 versus 1.7 months) and the objective response rates suggested some clinical activity of the bevacizumab plus erlotinib combination. In the aTLaS study, designed to evaluate the combination of bevacizumab with erlotinib versus bevacizumab alone in patients with stage IIIb/IV NSCLC, the primary endpoint of improving PFS was met (4.8 versus 3.7 months) and the safety profile for the combination was consistent with the profiles known for the two drugs (Kabbinavar et al., 2010).

In general, the results obtained so far from combinatorial EGFR-targeted therapy are not encouraging, except for the combination of bevacizumab and erlotinib in advanced NSCLC. as described above, the combination of bevacizumab with cetuximab or panitumumab showed deleterious effect in mCRC. Noteworthy, it has been suggested that cetuximab in mCRC may activate tumor promoting M2 macrophages (Pander et al., 2011), which in turn induce chronic inflammation in the tumor micro-environment, that would facilitate tumor progression (Mantovani et al., 2008).

### RATIONAL DESIGN OF COMBINATORIAL TARGETED THERAPIES SHOULD DRIVE DRUG DEVELOPMENT

Some stimulating results have recently been obtained in the preclinical setting with combinations of antibodies targeting one or two members of the ErbB family. Combinations of different, non-competitive antibodies targeting the EGFR (including cetuximab and panitumumab, but not together in the same combination since they compete with each other) inhibited tumor growth in triple-negative breast cancer models by promoting a more efficient down-regulation and degradation of the receptor (Ferraro et al., 2013). Likewise, combination of cetuximab with an anti-HER4 antibody and with radiotherapy was more effective in reducing cell survival and tumor growth using head and neck cancer cell lines (Barnea et al., 2013). Testing of any of these combinations in the clinic, however, is hampered by the fact that in all the assayed drug dyads at most only one of the antibodies is a registered therapeutic agent.

The clinical results obtained so far from combinatorial therapies reinforce the main idea discussed in the previous section that the oncogene addiction phenomenon may involve the interconnection of several cellular pathways. EGFR-targeted therapy might then modulate (either increase or decrease) the addiction of tumor cells to other oncogenes. Predicting whether a given combination will produce this phenomenon of trans-modulation of oncogene addiction is currently a very difficult task due the complexity of the intracellular signaling networks. a tempting approach involving targets that are not in cancer cells is to combine anti-EGFR agents with drugs that may enhance the immune system response against the tumor; for example, with an anti-cytotoxic T lymphocyte antigen 4 (anti-CTLa-4) antibody such as ipilimumab (Lipson and Drake, 2011).

Studying in the clinical setting the resistance mechanisms that emerge from the use of EGFR-targeted agents would provide important clues for the rational design of effective combinatorial therapies. Probably, some of the envisioned combinations will demand drugs that are not yet in the clinic, while some other designs will require totally new drugs. Thus, the quest for better combinatorial therapies would drive the development of new therapeutic agents with the express purpose of using them not alone, but in specific drug combinations.

## CONCLUDING REMARKS

Epidermal growth factor receptor-targeted therapies, although they have provided clinical benefit, have not completely fulfilled our expectations so far. a lesson from the available clinical data is that a new clinical research paradigm is required to evaluate targeted therapies. In particular, the concept of personalized medicine has not been yet translated to the design of pivotal clinical trials.

In our view, clinical investigation of EGFR-targeted therapies should follow these principles: (1) Treatment should be indicated based on an operational definition of EGFR oncogene addiction, which should be split according to clinical characteristics like tumor type, disease staging and previous treatments, as well as the existing knowledge on resistance mechanisms; (2) The design of combinatorial therapies that target, in addition to the EGFR, other oncogenes should be based on experimental and clinical evidences showing that these oncogenes play a role in the acquired resistance to the EGFR-targeting agent. This way, EGFR-targeted therapy would be translated into a set of treatment niches defined under the approach of “personalized medicine.”

## Conflict of Interest Statement

The authors declare that the research was conducted in the absence of any commercial or financial relationships that could be construed as a potential conflict of interest.

## References

[B1] abhold E. L. Kiang a. Rahimy E. Kuo S. Z. Wang-Rodriguez J. Lopez J. P. (2012). EGFR kinase promotes acquisition of stem cell-like properties: a potential therapeutic target in head and neck squamous cell carcinoma stem cells. *PLoS ONE * 7:e32459 10.1371/journal.pone.0032459 PMC3288103 22384257

[B2] aguiar alpizar Y. Karwacz K. arce F. Yglesias Rivera a. Fernández L. E. Collins M. K. (2012). Lentiviral vector followed by protein immunisation breaks tolerance against the self-antigen Her1 and results in lung cancer immunotherapy. *J. Gene Med.* 14 151–157 22262303 10.1002/jgm.2606

[B3] ayuso-Sacido a. Moliterno J. a. Kratovac S. Kapoor G. S. O’Rourke D. M. Holland E. C. (2010). activated EGFR signaling increases proliferation, survival, and migration and blocks neuronal differentiation in post-natal neural stem cells. *J. Neurooncol.* 97 323–337 19855928 10.1007/s11060-009-0035-x

[B4] Bao S. Wu Q. McLendon R. E. Hao Y. Shi Q. Hjelmeland a. B. (2006). Glioma stem cells promote radioresistance by preferential activation of the DNa damage response. *Nature* 444 756–760 17051156 10.1038/nature05236

[B5] Barnea I. Haif S. Keshet R. Karaush V. Lev ari S. Khafif a. (2013). Targeting ErbB-1 and ErbB-4 in irradiated head and neck cancer: results of in vitro and in vivo studies. *Head Neck* 35 399–407 22367849 10.1002/hed.22967

[B6] Basavaraj C. Sierra P. Shivu J. Melarkode R. Montero E. Nair P. (2010). Nimotuzumab with chemoradiation confersasurvival advantage in treatment-naïve head and neck tumors over expressing EGFR. *Cancer Biol. Ther.* 10 673–681 20647773 10.4161/cbt.10.7.12793

[B7] Bonner J. A. Harari P. M. Giralt J. Azarnia N. Shin D. M. Cohen R. B. (2006). Radiotherapy plus cetuximab for squamous-cell carcinoma of the head and neck. *N. Engl. J. Med.* 354 567–578 16467544 10.1056/NEJMoa053422

[B8] Califano R. Landi L. Cappuzzo F. (2012). Prognostic and predictive value of K-RAS mutations in non-small cell lung cancer. *Drugs* 72(Suppl.1) 28–36 22712795 10.2165/1163012-S0-000000000-00000

[B9] Cappuzzo F. Ciuleanu T. Stelmakh L. Cicenas S. Szczésna A. Juhász E. (2010). Erlotinib as maintenance treatment in advanced non-small-cell lung cancer:amulticentre, randomised, placebo-controlled phase 3 study. *Lancet Oncol.* 11 521–529 20493771 10.1016/S1470-2045(10)70112-1

[B10] Crombet T. Osorio M. Cruz T. Roca C. del Castillo R. Mon R. (2004). Use of the humanized anti-epidermal growth factor receptor monoclonal antibody h-R3 in combination with radiotherapy in the treatment of locally advanced head and neck cancer patients. *J. Clin. Oncol.* 22 1646–1654 15117987 10.1200/JCO.2004.03.089

[B11] Crombet-Ramos T. Rak J. Pérez R. Viloria-Petit A. (2002). Antiproliferative, antiangiogenic and proapoptotic activity of h-R3:ahumanized anti-EGFR antibody. *Int. J. Cancer* 101 567–575 12237899 10.1002/ijc.10647

[B12] Cunningham D. Humblet Y. Siena S. Khayat D. Bleiberg H. Santoro A. (2004). Cetuximab monotherapy and cetuximab plus irinotecan in irinotecan-refractory metastatic colorectal cancer. *N. Engl. J. Med.* 351 337–345 15269313 10.1056/NEJMoa033025

[B13] Del Vecchio C. A. Jensen K. C. Nitta R. T. Shain A. H. Giacomini C. P. Wong A. J. (2012). Epidermal growth factor receptor variant III contributes to cancer stem cell phenotypes in invasive breast carcinoma. *Cancer Res.* 72 2657–2671 22419663 10.1158/0008-5472.CAN-11-2656

[B14] Diaz Miqueli A. Rolff J. Lemm M. Fichtner I. Perez R. Montero E. (2009). Radiosensitization of U87MG brain tumours by anti-epidermal growth factor receptor monoclonal antibodies. *Br. J. Cancer* 100 950–958 19293809 10.1038/sj.bjc.6604943 PMC2661790

[B15] Downward J. Yarden Y. Mayes E. Scrace G. Totty N. Stockwell P. (1984). Close similarity of epidermal growth factor receptor and v-erb-B oncogene protein sequences. *Nature* 307 521–527 6320011 10.1038/307521a0

[B16] Driessens G. Beck B. Caauwe A. Simons B. D. Blanpain C. (2012). Defining the mode of tumour growth by clonal analysis. *Nature* 488 527–530 22854777 10.1038/nature11344 PMC5553110

[B17] El-Haibi C. P. Bell G. W. Zhang J. Collmann A. Y. Wood D. Scherber C. M. (2012). Critical role for lysyl oxidase in mesenchymal stem cell-driven breast cancer malignancy. *Proc. Natl. Acad. Sci. U.S.A.* 109 17460–17465 23033492 10.1073/pnas.1206653109 PMC3491529

[B18] Engelman J. A. (2009). Targeting PI3K signalling in cancer: opportunities, challenges and limitations. *Nat. Rev. Cancer* 9 550–562 19629070 10.1038/nrc2664

[B19] Engelman J. A. Zejnullahu K. Mitsudomi T. Song Y. Hyland C. Park J. O. (2007). MET amplification leads to gefitinib resistance in lung cancer by activating ERBB3 signaling. *Science* 316 1039–1043 17463250 10.1126/science.1141478

[B20] Eyler C. E. Foo W.-C. LaFiura K. M. McLendon R. E. Hjelmeland A. B. Rich J. N. (2008). Brain cancer stem cells display preferential sensitivity to Akt inhibition. *Stem Cells* 26 3027–3036 18802038 10.1634/stemcells.2007-1073 PMC2739007

[B21] Ferraro D. A. Gaborit N. Maron R. Cohen Dvashi H. Porat Z. Pareja F. (2013). Inhibition of triple-negative breast cancer models by combinations of antibodies to EGFR. *Proc. Natl. Acad. Sci. U.S.A.* 110 1815–1820 23319610 10.1073/pnas.1220763110 PMC3562774

[B22] Garrett J. T. Arteaga C. L. (2011). Resistance to HER2-directed antibodies and tyrosine kinase inhibitors: mechanisms and clinical implications. *Cancer Biol. Ther.* 11 793–800 21307659 10.4161/cbt.11.9.15045 PMC3230295

[B23] Garrido G. Lorenzano P. Sánchez B. Beausoleil I. Alonso D. F. Pérez R. (2007). T cells are crucial for the anti-metastatic effect of anti-epidermal growth factor receptor antibodies. *Cancer Immunol. Immunother.* 56 1701–1710 17415565 10.1007/s00262-007-0313-4 PMC11031102

[B24] Garrido G. Rabasa A. Sánchez B. López M. V. Blanco R. López A. (2011a). Induction of immunogenic apoptosis by blockade of epidermal growth factor receptor activation withaspecific antibody. *J. Immunol.* 187 4954–4966 21984704 10.4049/jimmunol.1003477

[B25] Garrido G. Tikhomirov I. A. Rabasa A. Yang E. Gracia E. Iznaga N. (2011b). Bivalent binding by intermediate affinity of nimotuzumab:acontribution to explain antibody clinical profile. *Cancer Biol. Ther.* 11 373–382 21150278 10.4161/cbt.11.4.14097

[B26] Guarino M. J. Schneider C. J. Hosford M. A. Brahmer J. R. Rudin C. M. Finckenstein F. G. (2009). Dual inhibition of the epidermal growth factor receptor pathway with cetuximab and erlotinib:aphase I study in patients with advanced solid malignancies. *Oncologist* 14 119–124 19182243 10.1634/theoncologist.2008-0124 PMC5310714

[B27] Guo G. F. Cai Y. C. Zhang B. Xu R. H. Qiu H. J. Xia L. P. (2011). Overexpression of SGLT1 and EGFR in colorectal cancer showingacorrelation with the prognosis. *Med. Oncol.* 28(Suppl.1) S197–S203 21080109 10.1007/s12032-010-9696-8

[B28] Hanabata Y. Nakajima Y. Morita K. Kayamori K. Omura K. (2012). Coexpression of SGLT1 and EGFR is associated with tumor differentiation in oral squamous cell carcinoma. *Odontology* 100 156–163 21607591 10.1007/s10266-011-0033-2

[B29] Hardy K. M. Booth B. W. Hendrix M. J. C. Salomon D. S. Strizzi L. (2010). ErbB/EGF signaling and EMT in mammary development and breast cancer. *J. Mammary Gland Biol. Neoplasia* 15 191–199 20369376 10.1007/s10911-010-9172-2 PMC2889136

[B30] Hendler F. J. Ozanne B. W. (1984). Human squamous cell lung cancers express increased epidermal growth factor receptors. *J. Clin. Invest.* 74 647–651 6086719 10.1172/JCI111463 PMC370518

[B31] Herbst R. S. Ansari R. Bustin F. Flynn P. Hart L. Otterson G. A. (2011). Efficacy of bevacizumab plus erlotinib versus erlotinib alone in advanced non-small-cell lung cancer after failure of standard first-line chemotherapy (BeTa):adouble-blind, placebo-controlled, phase 3 trial. *Lancet* 377 1846–1854 21621716 10.1016/S0140-6736(11)60545-X PMC4134127

[B32] Huber S. M. Misovic M. Mayer C. Rodemann H.-P. Dittmann K. (2012). EGFR-mediated stimulation of sodium/glucose cotransport promotes survival of irradiated human A549 lung adenocarcinoma cells. *Radiother. Oncol.* 103 373–379 22516777 10.1016/j.radonc.2012.03.008

[B33] Janjigian Y. Y. Azzoli C. G. Krug L. M. Pereira L. K. Rizvi N. A. Pietanza M. C. (2011). Phase I/II trial of cetuximab and erlotinib in patients with lung adenocarcinoma and acquired resistance to erlotinib. *Clin. Cancer Res.* 17 2521–2527 21248303 10.1158/1078-0432.CCR-10-2662

[B34] Jedlinski A. Ansell A. Johansson A. C. Roberg K. (2013). EGFR status and EGFR ligand expression influence the treatment response of head and neck cancer cell lines. *J. Oral Pathol. Med.* 42 26–36 22643066 10.1111/j.1600-0714.2012.01177.x

[B35] Jonker D. J. O’Callaghan C. J. Karapetis C. S. Zalcberg J. R. Tu D. Au H.-J. (2007). Cetuximab for the treatment of colorectal cancer. *N. Engl. J. Med.* 357 2040–2048 18003960 10.1056/NEJMoa071834

[B36] Kabbinavar F. F. Miller V. A. Johnson B. E. O’Connor P. G. Soh P. G ATLAS Investigators (2010). Overall survival (OS) in ATLAS,aphase IIIb trial comparing bevacizumab (B) therapy with or without erlotinib (E) after completion of chemotherapy (chemo) with B for first-line treatment of locally advanced, recurrent, or metastatic non-small cell lung cancer (NSCLC). *J. Clin. Oncol.* 28(Suppl. 15),abstr 7526

[B37] Khambata-Ford S. Garrett C. R. Meropol N. J. Basik M. Harbison C. T. Wu S. (2007). Express- ion of epiregulin and amphiregulin and K-ras mutation status predict disease control in metastatic colo- rectal cancer patients treated with cetuximab. *J. Clin. Oncol.* 25 3230–3237 17664471 10.1200/JCO.2006.10.5437

[B38] Kim Y. H. Sasaki Y. Lee K. H. Rha S. Y. Park S. H. Boku N. (2011). Randomized phase II study of nimotuzumab, an anti-EGFR antibody, plus irinotecan in patients with 5-fluorouracil-based regimen-refractory advanced or recurrent gastric cancer in Korea and Japan: preliminary results. *J. Clin. Oncol.* 29(Suppl. 4),abstr. 87

[B39] Kroemer G. Pouyssegur J. (2008). Tumor cell metabolism: cancer’s Achilles’ heel. *Cancer Cell* 13 472–482 18538731 10.1016/j.ccr.2008.05.005

[B40] Lièvre A. Bachet J. B. Le Corre D. Boige V. Landi B. Emile J. F. (2006). KRAS mutation status is predictive of response to cetuximab therapy in colorectal cancer. *Cancer Res.* 66 3992–3995 16618717 10.1158/0008-5472.CAN-06-0191

[B41] Lipson E. J. Drake C. G. (2011). Ipilimumab: an anti-CTLA-4 antibody for metastatic melanoma. *Clin. Cancer Res.* 17 6958–6962 21900389 10.1158/1078-0432.CCR-11-1595 PMC3575079

[B42] Luedke E. Jaime-Ramirez A. C. Bhave N. Roda J. Choudhary M. M. Kumar B. (2012). Cetuximab therapy in head and neck cancer: immune modulation with interleukin-12 and other natural killer cell-activating cytokines. *Surgery* 152 431–440 22770960 10.1016/j.surg.2012.05.035 PMC3432674

[B43] Mantovani A. Allavena P. Sica A. Balkwill F. (2008). Cancer-related inflammation. *Nature* 454 436–444 18650914 10.1038/nature07205

[B44] Mateo C. Moreno E. Amour K. Lombardero J. Harris W Pérez R. (1997). Humanization ofamouse monoclonal antibody that blocks the epidermal growth factor receptor: recovery of antagonistic activity. *Immunotechnology* 3 71–81 9154469 10.1016/s1380-2933(97)00065-1

[B45] Mazzoleni S. Politi L. S. Pala M. Cominelli M. Franzin A. Sergi Sergi L. (2010). Epidermal growth factor receptor expression identifies functionally and molecularly distinct tumor-initiating cells in human glioblastoma multiforme and is required for gliomagenesis. *Cancer Res.* 70 7500–7513 20858720 10.1158/0008-5472.CAN-10-2353

[B46] Montagut C. Dalmases A. Bellosillo B. Crespo M. Pairet S. Iglesias M. (2012). Identification ofamutation in the extracellular domain of the epidermal growth factor receptor conferring cetuximab resistance in colorectal cancer. *Nat. Med.* 18 221–223 22270724 10.1038/nm.2609

[B47] Moore M. J. Goldstein D. Hamm J. Figer A. Hecht J. R. Gallinger S. (2007). Erlotinib plus gemcitabine compared with gemcitabine alone in patients with advanced pancreatic cancer:aphase III trial of the National Cancer Institute of Canada Clinical Trials Group. *J. Clin. Oncol.* 25 1960–1966 17452677 10.1200/JCO.2006.07.9525

[B48] Nguyen L. V. Vanner R. Dirks P. Eaves C. J. (2012). Cancer stem cells: an evolving concept. *Nat. Rev. Cancer* 12 133–143 22237392 10.1038/nrc3184

[B49] Pander J. Heusinkveld M. Van der Straaten T. Jordanova E. S. Baak-Pablo R. Gelderblom H. (2011). Activation of tumor-promoting type 2 macrophages by EGFR-targeting antibody cetuximab. *Clin. Cancer Res.* 17 5668–5673 21788356 10.1158/1078-0432.CCR-11-0239

[B50] Pao W. Miller V. A. Politi K. A. Riely G. J. Somwar R. Zakowski M. F. (2005). Acquired resistance of lung adenocarcinomas to gefitinib or erlotinib is associated withasecond mutation in the EGFR kinase domain. *PLoS Med.* 2:e73 10.1371/journal.pmed.0020073 PMC549606 15737014

[B51] Perez R. Moreno E. Garrido G. Crombet T. (2011). EGFR-targeting asabiological therapy: understanding Nimotuzumab’s clinical effects. *Cancers* 3 2014–2031 10.3390/cancers3022014 PMC3757402 24212794

[B52] Pérol M. Chouaid C. Pérol D. Barlési F. Gervais R. Westeel V. (2012). Randomized, phase III study of gemcitabine or erlotinib maintenance therapy versus observation, with predefined second-line treatment, after cisplatin-gemcitabine induction chemotherapy in advanced non-small-cell lung cancer. *J. Clin. Oncol.* 30 3516–3524 22949150 10.1200/JCO.2011.39.9782

[B53] Pirker R. Pereira J. R. Szczesna A. Von Pawel J. Krzakowski M. Ramlau R. (2009). Cetuximab plus chemotherapy in patients with advanced non-small-cell lung cancer (FLEX): an open-label randomised phase III trial. *Lancet* 373 1525–1531 19410716 10.1016/S0140-6736(09)60569-9

[B54] Pirker R. Pereira J. R. Von Pawel J. Krzakowski M. Ramlau R. Park K. (2012). EGFR expression asapredictor of survival for first-line chemotherapy plus cetuximab in patients with advanced non-small-cell lung cancer: analysis of data from the phase 3 FLEX study. *Lancet Oncol.* 13 33–42 22056021 10.1016/S1470-2045(11)70318-7

[B55] Quatrale A. E. Porcelli L. Silvestris N. Colucci G. Angelo A. Azzariti A. (2011). EGFR tyrosine kinases inhibitors in cancer treatment: in vitro and in vivo evidence. *Front. Biosci.* 16 1962–1972 21196276 10.2741/3833

[B56] Rakhra K. Bachireddy P. Zabuawala T. Zeiser R. Xu L. Kopelman A. (2010). CD4(+) T cells contribute to the remodeling of the microenvironment required for sustained tumor regression upon oncogene inactivation. *Cancer Cell* 18 485–498 21035406 10.1016/j.ccr.2010.10.002 PMC2991103

[B57] Ramírez B. S. Alpízar Y. A. Fernández D. R. H. Hidalgo G. G. Capote A. R. Rodríguez R. P. (2008). Anti-EGFR activation, anti-proliferative and pro-apoptotic effects of polyclonal antibodies induced by EGFR-based cancer vaccine. *Vaccine* 26 4918–4926 18672015 10.1016/j.vaccine.2008.07.018

[B58] Ramírez B. S. Pestana E. S. Hidalgo G. G. García T. H. Rodríguez R. P. Ullrich A. (2006). Active antimetastatic immunotherapy in Lewis lung carcinoma with self EGFR extracellular domain protein in VSSP adjuvant. *Int. J. Cancer* 119 2190–2199 16841332 10.1002/ijc.22085

[B59] Ramos-Suzarte M. Lorenzo-Luaces P. Lazo N. G. Perez M. L. Soriano J. L. Gonzalez C. E. V. (2012). Treatment of malignant, non-resectable, epithelial origin esophageal tumours with the humanized anti-epidermal growth factor antibody nimotuzumab combined with radiation therapy and chemotherapy. *Cancer Biol. Ther.* 13 600–605 22555809 10.4161/cbt.19849

[B60] Rodrïguez M. O. Rivero T. C. Del Castillo Bahi R. Muchuli C. R. Bilbao M. A. Vinageras E. N. (2010). Nimotuzumab plus radiotherapy for unresectable squamous-cell carcinoma of the head and neck. *Cancer Biol. Ther.* 9 343–349 20448462 10.4161/cbt.9.5.10981

[B61] Rosell R. Moran T. Queralt C. Porta R. Cardenal F. Camps C. (2009). Screening for epidermal growth factor receptor mutations in lung cancer. *N. Engl. J. Med.* 361 958–967 19692684 10.1056/NEJMoa0904554

[B62] Rosell R. Taron M. Sanchez J. J. Paz-Ares L. (2007). Setting the benchmark for tailoring treatment with EGFR tyrosine kinase inhibitors. *Future Oncol.* 3 277–283 17547522 10.2217/14796694.3.3.277

[B63] Saltz L. Badarinath S. Dakhil S. Bienvenu B. Harker W. G. Birchfield G. (2012). Phase III trial of cetuximab, bevacizumab, and 5-fluorouracil/leucovorin vs. FOLFOX-bevacizumab in colorectal cancer. *Clin. Colorectal Cancer* 11 101–111 22055112 10.1016/j.clcc.2011.05.006

[B64] Sato A. Sunayama J. Matsuda K. Tachibana K. Sakurada K. Tomiyama A. (2010). Regulation of neural stem/progenitor cell maintenance by PI3K and mTOR. *Neurosci. Lett.* 470 115–120 20045038 10.1016/j.neulet.2009.12.067

[B65] Saurez G. Cabanas R. Zaldívar M. Garnier T. Iglesias B. Piedra P. (2009). Clinical experience with nimotuzumab in cuban pediatric patients with brain tumors, 2005 to 2007. *MEDICC Rev.* 11 27–33 21483304 10.37757/MR2009V11.N3.7

[B66] Schmitz K. R. Ferguson K. M. (2009). Interaction of antibodies with ErbB receptor extracellular regions. *Exp. Cell Res.* 315 659–670 18992239 10.1016/j.yexcr.2008.10.008 PMC2726996

[B67] Shepherd F. A. Rodrigues Pereira J. Ciuleanu T. Tan E. H. Hirsh V. Thongprasert S. (2005). Erlotinib in previously treated non-small-cell lung cancer. *N. Engl. J. Med.* 353 123–132 16014882 10.1056/NEJMoa050753

[B68] Sporn M. B. Todaro G. J. (1980). Autocrine secretion and malignant transformation of cells. *N. Engl. J. Med.* 303 878–880 7412807 10.1056/NEJM198010093031511

[B69] Talavera A. Friemann R. Gómez-Puerta S. Martinez-Fleites C. Garrido G. Rabasa A. (2009). Nimotuzumab, an antitumor antibody that targets the epidermal growth factor receptor, blocks ligand binding while permitting the active receptor conformation. *Cancer Res.* 69 5851–5859 19584289 10.1158/0008-5472.CAN-08-4518

[B70] Tol J. Koopman M. Cats A. Rodenburg C. J. Creemers G. J. M. Schrama J. G. (2009). Chemotherapy, bevacizumab, and cetuximab in metastatic colorectal cancer. *N. Engl. J. Med.* 360 563–572 19196673 10.1056/NEJMoa0808268

[B71] Ullrich A. Coussens L. Hayflick J. S. Dull T. J. Gray A. Tam A. W. (1984). Human epidermal growth factor receptor cDNA sequence and aberrant expression of the amplified gene in A431 epidermoid carcinoma cells. *Nature* 309 418–425 6328312 10.1038/309418a0

[B72] Van Cutsem E. Köhne C.-H. Hitre E. Zaluski J. Chang Chien C.-R. Makhson A. (2009). Cetuximab and chemotherapy as initial treatment for metastatic colorectal cancer. *N. Engl. J. Med.* 360 1408–1417 19339720 10.1056/NEJMoa0805019

[B73] Vermorken J. B. Herbst R. S. Leon X. Amellal N. Baselga J. (2008). Overview of the efficacy of cetuximab in recurrent and/or metastatic squamous cell carcinoma of the head and neck in patients who previously failed platinum-based therapies. *Cancer* 112 2710–2719 18481809 10.1002/cncr.23442

[B74] Viloria-Petit A. Crombet T. Jothy S. Hicklin D. Bohlen P. Schlaeppi J. M. (2001). Acquired resistance to the antitumor effect of epidermal growth factor receptor-blocking antibodies in vivo:arole for altered tumor angiogenesis. *Cancer Res.* 61 5090–5101 11431346

[B75] Weihua Z. Tsan R. Huang W.-C. Wu Q. Chiu C.-H. Fidler I. J. (2008). Survival of cancer cells is maintained by EGFR independent of its kinase activity. *Cancer Cell* 13 385–393 18455122 10.1016/j.ccr.2008.03.015 PMC2413063

[B76] Weinstein I. B. (2002). Cancer. Addiction to oncogenes – the Achilles heal of cancer. * Science* 297 63–64 12098689 10.1126/science.1073096

[B77] Weinstein I. B. Joe A. K. (2006). Mechanisms of disease: Oncogene addiction –arationale for molecular targeting in cancer therapy. *Nat. Clin. Pract. Oncol.* 3 448–457 16894390 10.1038/ncponc0558

[B78] Weinstein I. B. Joe A. K. (2008). Oncogene addiction. *Cancer Res.* 68 3077–3080 18451130 10.1158/0008-5472.CAN-07-3293

[B79] Weiss G. J. Bemis L. T. Nakajima E. Sugita M. Birks D. K. Robinson W. A. (2008). EGFR regulation by microRNA in lung cancer: correlation with clinical response and survival to gefitinib and EGFR expression in cell lines. *Ann. Oncol.* 19 1053–1059 18304967 10.1093/annonc/mdn006

[B80] Wright E. M. Loo D. D. F. Hirayama B. A. (2011). Biology of human sodium glucose transporters. *Physiol. Rev.* 91 733–794 21527736 10.1152/physrev.00055.2009

[B81] Yan X. Fu C. Chen L. Qin J. Zeng Q. Yuan H. (2012). Mesenchymal stem cells from primary breast cancer tissue promote cancer proliferation and enhance mammosphere formation partially via EGF/EGFR/Akt pathway. *Breast Cancer Res. Treat.* 132 153–164 21584665 10.1007/s10549-011-1577-0

[B82] Yarden Y. Pines G. (2012). The ERBB network: at last, cancer therapy meets systems biology. *Nat. Rev. Cancer* 12 553–563 22785351 10.1038/nrc3309

[B83] Yonesaka K. Zejnullahu K. Okamoto I. Satoh T. Cappuzzo F. Souglakos J. (2011). Activation of ERBB2 signaling causes resistance to the EGFR-directed therapeutic antibody cetuximab. *Sci. Transl. Med.* 3 99ra86 10.1126/scitranslmed.3002442 PMC3268675 21900593

[B84] Yoshikawa S. Kukimoto-Niino M. Parker L. Handa N. Terada T. Fujimoto T. (2013). Structural basis for the altered drug sensitivities of non-small cell lung cancer-associated mutants of human epidermal growth factor receptor. *Oncogene* 32 27–38 22349823 10.1038/onc.2012.21

[B85] Zhang D. LaFortune T. A. Krishnamurthy S. Esteva F. J. Cristofanilli M. Liu P. (2009). Epidermal growth factor receptor tyrosine kinase inhibitor reverses mesenchymal to epithelial phenotype and inhibits metastasis in inflammatory breast cancer. *Clin. Cancer Res.* 15 6639–6648 19825949 10.1158/1078-0432.CCR-09-0951 PMC2783487

